# Frequency of Sports Trauma in Elite National Level Greco-Roman Wrestling Competitions

**DOI:** 10.5812/atr.6866

**Published:** 2012-08-21

**Authors:** Ali Akbarnejad, Mansour Sayyah

**Affiliations:** 1 College of Physical Education, University of Tehran, Tehran, IR Iran; 2 Trauma Research Center, Kashan University of Medical Sciences, Kashan, IR Iran

**Keywords:** Sports, Wound and Injuries, Wrestling

## Abstract

**Background:**

Trauma is an inescapable part of sports competitions. It occurs more frequently in contact sports such as wrestling.

**Objectives:**

The purpose of this study was to determine the frequency of injury in Greco-Roman style wrestling competitions at national level.

**Patients and Methods:**

This descriptive epidemiological research included 50 Greco-Roman style wrestlers who participated in national level competitions between the years 2003 and 2008. A questionnaire was completed by each participant, itincluded; the number of injuries to skin, muscle tissue, bones and joints. The reliability of the instrument was evaluated by a test – retest method (r = 0.83, P = 0.0001).

**Results:**

The most frequent injuries encountered by the wrestlers were; skin lesions (62%), followed by muscle (22%), bone (9%) and joint (7%) injuries, respectively.

**Conclusions:**

Greco-Roman style wrestlers are at high risk of skin injuries.Therefore, they need appropriate instructions on how to avoid injuries and adequate care after their competitions.

## 1. Background

Wrestling is a popular sport in many countries around the world. Its origins can be traced back to the Sumerians as early as 5000 BC, and records of ancient Greeks wrestling in the Olympics in 708 B.C. (1). This sport was played without any particular rules prior to the Olympic movement (2). Wrestling is the national sport of Iran and quite popular across the country (3-5). Wrestling has been referred to as the most intense and physically demanding sport, with a high risk of injury. This sport has evolved into many different forms all over the world, with three major styles including; freestyle, Greco-Roman and folk styles. In the United States, wrestling participation averaged 2.5 million participants per year between 2000-2006, with an average of 1.1 million participants wrestling more than 50 days per year (6). In this sport, the two athletes are expected to struggle together and endeavor to put their competitor down onto the mat. There are two different rules that distinguish freestyle wrestling from its Greco-Roman counterpart. In the Greco-Roman style, athletes are forbidden to touch the lower extremities of their rivals, while this is not so in the freestyle. However, in both types, the athletes must struggle extremely hard to gain dominance over the strength of their rival and at the same time try to force their opponent to give up their defense once he/she is down on the mat. In this competition all parts of the body are involved in voluntary movements at times and involuntary movements at others, which are directed at overcoming the resistance and strength of the rival wrestler. During this process, biomechanical forces are imposed on both athletes, which in some circumstances may lead to undesirable consequences, including injury to different parts of the body.(1). According to data from the Center for Injury Research and Policy, football and wrestling are the two sports with the highest risk of serious injury to athletes (7-9). Considering the specific types of injury; sprains, strains, and contusions were the most commonly reported types (8). In a study conducted by Jarret et al. on 800000 athletes participating in college level competitions in the United States over 11 years, they reported that 6.3% of the trauma cases that took place in the competitions needed surgical intervention. In addition, the study showed that; sprains, strains, contusions and muscle pulls were the most frequent types of injuries, respectively (8). A study on wrestling injuries that occurred during the 2008 Olympic Games in Beijing, showed a total of 32 injuries occurred in 343 athletes during the 406 matches, which is equivalent to an overall incidence rate of 9.3 injuries per 100 athletes and 7.9 injuries per 100 matches. Among the 2 styles, freestyle had the highest injury rate (10.1%), and female wrestling the lowest rate (7.5%). The overall injury rate in the male athletes was slightly higher than in the females (9.7% versus 7.5%) (10).

## 2. Objectives

This research was designed to assess Iranian elite national level Greco-Roman wrestlers, in regard to their experience of injury during their careers as wrestlers.

## 3. Patients and Methods

This descriptive study included 50 former Greco-Roman style wrestlers competing at national level in Iran. The criteria to enroll these elite athletes in this study included an invitation by the National Wrestling Federation for the athlete to attend a preparation camp, and record of the athlete’s participation in national level competitions, Asian Games, world championship tournaments and/or the Olympic Games. The data collection form included questions regarding demographic information as well as the incidence of injury during their competitions. The content validity of the questionnaire was confirmed by expert opinion, including former wrestlers and university professors involved in teaching sport trauma in physical education and exercise science colleges. The reliability of the instrument was examined by a test-retest of 20 of the wrestlers participating in this project (r = 0.83, P = 0.0001). All of the subjects completed the final version of the questionnaire in the camp where the researcher was available to give further instructions, if it was requested.

## 4. Results

**Figure 1:**
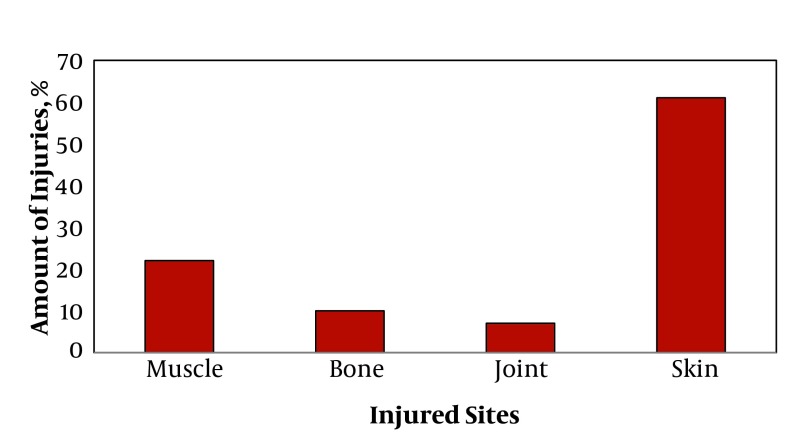
Frequency Distribution of Injuries in National Level Greco-Roman Wrestlers According to the Tissues Involved

A total of 50 national level Greco-Roman style wrestlers completed the study criteria. Mean age of the participants was 22.2 ± 2.4 year, weight 81.2 ± 9.8 kg and height 178 ± 3.7cm, respectively. Analysis of the data showed 6514 cases of injuries overall during the 5 year peak of the athletes’ wrestling career. Out of 6514 injuries, 3794 (62%) of the cases reported injuries to their skin, followed by muscle tissue 1448 (22%). Figure 1 shows the sites of injuries.

Further analysis showed that most of the injuries happened in the upper extremities, followed by the trunk and spinal regions. Table 1 presents a distribution of injury cases on the basis of the tissues which were involved. In addition, a one-way analysis of variance (ANOVA) was performed to compare the mean value of injuries from the four different types of injuries, that is; skin, muscle, bone and joints. The results indicated that there was a significant difference among the four types of injuries found in Greco-Roman style wrestlers (P < 0.0001). A Turkey’s post hoc test showed differences between the skin and other tissues (P < 0.0001). In addition, there was a significant difference found between muscle tissue injuries and bone and joint injuries (P < 0.001). No significant difference was found between joint and bone injuries (P > 0.05).

**Table 1.** Frequency Distribution of the Type of Injuries According to the Body Part Involved.

	Skin, No. (%)	Muscle, No. (%)	Bone, No. (%)	Joint, No. (%)	Total, No. (%)
Head and face	1253 (31)	-	364 (57)	77 (18)	1694 (26)
Upper extremities	1484 (37)	449 (31)	101 (16)	133 (30)	2167 (33.3)
Spine and trunk	982 (25)	660 (45)	144 (22)	60 (14)	1846 (28.4)
Lower extremities	277 (7)	339 (24)	25 (5)	165 (38)	807 (12.3)
Total	3996 (62)	1448 (22)	635 (9)	435 (7)	6514 (100)

## 5. Discussion

The present study showed that the highest incidence of injuries were to the athletes’ skin (62%), followed by muscle (22%), bones (9%) and joints (7%), respectively. These results were compatible with what has been reported earlier by other researchers (11). Considering the region of injury involvement, it was found that 33.3% of the injuries occurred in the upper extremities. Such findings are to be expected, since in Greco-Roman style wrestling, the wrestlers are restricted to competing with their counterpart only in this area, so most of the force is concentrated in the upper extremities, particularly in the hand itself. In a study by Snook, the upper limbs were the most commonly injured region (12). Also in a study carried out by Lorish et al. in adolescent and preadolescent boys, the primary areas of injury were to the upper extremities (33%), and the neck and back (24%) (2). Such findings can be attributed to the fact that in Greco-Roman style wrestling, most of the techniques executed in order to bring the opponent down and defeat him, involve the trunk. The consequence of such mechanical action on the opponent body will be to crash fall onto the mat, with the possible likelihood of injurious involvement of the skin, followed by extraordinary pressure on the trunk. In addition, following the landing on the mat, additional pressure is exerted to the trunk by rubbing the body on the mat, which may lead to simultaneous injuries of the skin, neck and trunk, as well. Other types of injuries reported in this study may affect other parts of the body, e.g., joints and bones, these may be due to a fall, as well as to impact forces at the time of collision between the two wrestlers .Injuries may also occur during the occasions when a wrestler collapses involuntarily over his opponent, this may cause dislocation of the joints or more seriously, breaking of bones. This has been the subject of many investigations by different authors (13, 14). In conclusion, wrestling in general as with any other type of sports, has its own risk of injury. However, the high incidence of sports trauma in wrestling requires special attention and good preparation by the athletes on one hand and awareness of appropriate care and support by sports authorities, trainers and coaches, on the other.
